# A New Method for Extraction and Analysis of Ricin Samples through MALDI-TOF-MS/MS

**DOI:** 10.3390/toxins11040201

**Published:** 2019-04-03

**Authors:** Roberto B. Sousa, Keila S. C. Lima, Caleb G. M. Santos, Tanos C. C. França, Eugenie Nepovimova, Kamil Kuca, Marcos R. Dornelas, Antonio L. S. Lima

**Affiliations:** 1 Brazilian Army CBRN Defense Institute (IDQBRN), Avenida das Américas 28705, Rio de Janeiro 23020-470, Brazil; rbtsousa@gmail.com; 2 Army Institute of Biology (IBEx), Rua Francisco Manuel, 102, Rio de Janeiro 20911-270, Brazil; keila@ime.eb.br (K.S.C.L.); calebguedes@gmail.com (C.G.M.S.); dornelas-ribeiro@hotmail.com (M.R.D.); 3 Laboratory of Molecular Modeling Applied to Chemical and Biological Defense, Military Institute of Engineering, Praca General Tiburcio 80, Rio de Janeiro 22290-270, Brazil; tanosfranca@gmail.com; 4 Department of Chemistry, Faculty of Science, University of Hradec Kralove, Rokitanskeho 62, 50003 Hradec Kralové, Czech Republic; eugenie.nepovimova@uhk.cz; 5 Chemical Engineering Department, Military Institute of Engineering, Praca General Tiburcio 80, Rio de Janeiro 22290-270, Brazil

**Keywords:** ricin, MALDI-TOF MS, chemical weapons, biological weapons, CBRN defense

## Abstract

We report for the first time the efficient use of accelerated solvent extraction (ASE) for extraction of ricin to analytical purposes, followed by the combined use of sodium dodecyl sulfate-polyacrylamide gel electrophoresis (SDS-PAGE), Matrix-assisted laser desorption/ionization time-of-flight mass spectrometry (MALDI-TOF MS), and MALDI-TOF MS/MS method. That has provided a fast and unambiguous method of ricin identification for in real cases of forensic investigation of suspected samples. Additionally, MALDI-TOF MS was applied to characterize the presence and the toxic activity of ricin in irradiated samples. Samples containing ricin were subjected to ASE, irradiated with different dosages of gamma radiation, and analyzed by MALDI-TOF MS/MS for verification of the intact protein signal. For identification purposes, samples were previously subjected to SDS-PAGE, for purification and separation of the chains, followed by digestion with trypsin, and analysis by MALDI-TOF MS/MS. The results were confirmed by verification of the amino acid sequences of some selected peptides by MALDI-TOF MS/MS. The samples residual toxic activity was evaluated through incubation with a DNA substrate, to simulate the attack by ricin, followed by MALDI-TOF MS/MS analyses.

## 1. Introduction

Ricin is a highly toxic protein which can be extracted from the castor bean seeds (*Ricinus communis* L.). This plant is present in all Brazilian regions and explored commercially for its oil, which is mainly used for the production of lubricants, fuel and drugs. Currently, Brazil is the fourth world producer of castor bean oil, just behind India, China and Mozambique [1,2]. The production of 1.0 ton of oil generates around 1.2 ton of residue, known as castor cake [3]. The literature reports different values for the ricin content in the castor cake, varying between 0.04% and 0.08% (w/w), depending on the cultivars, the extraction method, and the analysis [4,5,6]. Castor cake is an excellent source of nutrients for cattle; however, its content of ricin can intoxicate the animals. In addition, the disposal of this residue in the environment represents a risk for the population. The detoxification methods proposed so far for the castor cake are expensive, time and energy demanding, and do not guarantee the total destruction of ricin without formation of other toxic products. The analyses are usually based on oral toxicity and other experiments with animals that can be influenced by several factors like species, age, and feeding time. Spectrometric techniques for identification and quantification of the products formed have rarely been used for these studies [7,8].

Ricin has toxicity similar to the neurotoxic agent sarin and can be easily extracted from the castor bean (*R. communis* L.) seeds as a fine white powder, water soluble, and stable at a large range of pH. For this reason, it is considered a chemical/biological warfare agent scheduled by both the chemical weapons convention (CWC) [9], and the biological weapons convention (BWC) [10]. It can be disseminated in the air as fine particles with a diameter smaller than 5 microns or used to contaminate water supplies or agricultural products. This turns ricin into a perfect agent for terrorist attacks and a matter of big concern for national authorities worldwide [11,12,13,14].

The structure of the ricin molecule is made up of two different chains, named RTA and RTB, connected by a disulfide bond. RTA is an N-glycosidase containing 267 amino acids arranged in eight α-helices and eight β-strands, distributed in three structural domains, forming a “U” shaped cleft containing the protein active site. RTB is a lecithin composed of 262 amino acids, containing neither α-helices nor β-strands [12,15,16]. Due to its mechanism of action in the organism, and for being a heterodimer, ricin is classified as a ribosome inactivating protein (RIP) of type II [16,17,18]. RTB is responsible for the binding of ricin to the terminal galactose residues of the glycolipids and glycoproteins present on the surface of eukaryotic cells [12]. This enables the formation of a vesicle surrounding the toxin, which guides it into the inner part of the cell through endocytosis. Once inside the endosome, many ricin molecules are transported back to the outside of the cell or for the lysosomes, where they are degraded. However, some of them manage to reach the Golgi complex, following in retrograde movement, until the endoplasmic reticulum, where their disulfide bonds are cleaved, splitting RTA and RTB. After, RTA is transferred to the cytosol where it reacts specifically with the ribosomal RNA (rRNA) 28S of the ribosomal subunit 60S, provoking the hydrolysis of the N-glycoside bond of the adenine residue at position 4324 (A4324) [13,19,20,21]. 

The current decontamination process of people exposed to ricin consists only on the removal of clothes, followed by washing the skin with running water [11]. In cases of ingestion, the patient should be immediately submitted to gastrointestinal lavage [22]. There is no specific antidote for poisoning with ricin yet, neither a commercial vaccine [22,23]. Many works have been performed in the last decade towards the development of a vaccine, including tests with humans. Results have been promising, however, no final product has been approved yet [22,23,24,25,26].

The most common methods used for detection of ricin are based on enzyme-linked immunosorbent assay, like the ELISA method, or in bioassays where the inactivation of an RNA substrate is measured. Other techniques also used are sodium dodecyl sulfate-polyacrylamide gel electrophoresis (SDS-PAGE), real-time quantitative-polymerase chain reaction (RTQ-PCR), and toxicological analyses in cell culture and in guinea pigs [7,27,28,29,30,31,32,33]. In this work, we report for the first time the accelerated solvent extraction (ASE) as an efficient method for ricin extraction from the seeds of castor bean (*R. communis* L.) followed by the combined use of SDS-PAGE, matrix-assisted laser desorption/ionization/time-of-flight mass spectrometry (MALDI-TOF MS) and MALDI-TOF MS/MS for a fast and unambiguous identification of ricin for forensic purposes. Additionally, this method was further successfully used to detect the presence of ricin in gamma-irradiated samples.

## 2. Results and Discussion

### 2.1. Detection of the Intact Ricin Molecule by MALDI-TOF MS

**Figure 1:**
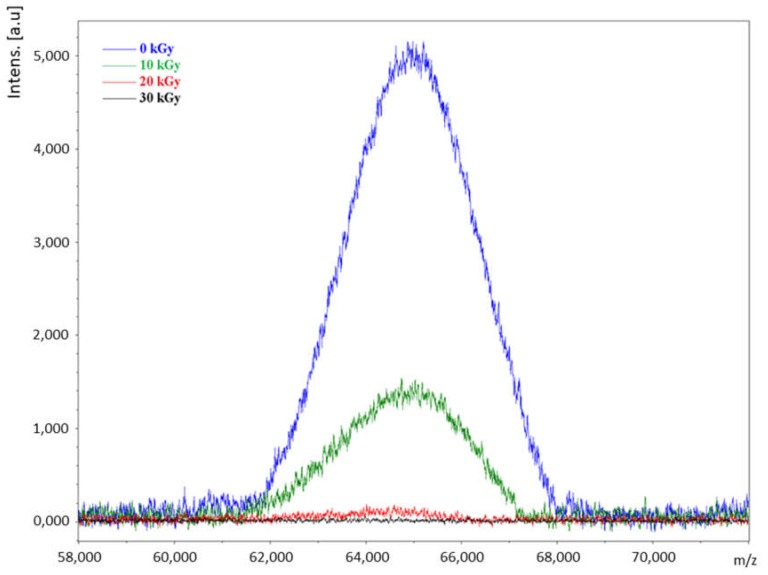
MALDI-TOF MS spectra of the non-irradiated ricin sample (0 kGy) and the samples irradiated at 10, 20 and 30 kGy.

Direct analysis of the samples through MALDI-TOF MS, led to a rapid identification of the characteristic peak of the intact molecule of ricin at *m*/*z* close to 64 kD, as shown in Figure 1. The exact position of this peak can change according to the simultaneous existence of different isoforms. The non-irradiated sample (0 kGy) showed a peak relatively intense in this region of the spectra (Figure 1), while the irradiated samples showed a reduction in intensity of this peak from 5000 a.u (non-irradiated sample) to around 1500 a.u (for sample irradiated at 10 kGy), and close to 0,000 a.u (for samples irradiated at 20 and 30 kGy). Table 1 reports the ratio signal/noise (S/N) for each sample, considering the triplicates analyzed. As can be seen, the irradiation dosage of 10, 20 and 30 kGy provoked average reductions of the signal to noise (S/N) of 65.9%, 91.8% and 97.5%, respectively, compared to the non-irradiated sample.

Direct analysis through MALDI-TOF MS showed useful for verification of the integrity of the ricin molecules present in the samples. The presence of a peak in the range between *m*/*z* 62,000 and 68,000 should be considered as an initial detection, indicating, in a semi-quantitative way, the possible presence of non-degraded ricin. This method has the advantage of being relatively rapid.

### 2.2. Unequivocal Identification of Ricin

According to the procedures adopted by the Organization for Prohibition of Chemical Weapons (OPCW) [9], the unequivocal identification of a chemical substance related to the CWC should be made through two different analytical methods, and one of them must be spectrometric. In order to meet this criterion, the identification of the ricin presence in the samples was performed through MALDI-TOF MS, using of peptide mass fingerprint (PMF) method after extraction of the protein separated through SDS-PAGE. Confirmation of the results was done with MALDI-TOF MS/MS.

#### 2.2.1. SDS-PAGE in Reducing Conditions

**Figure 2:**
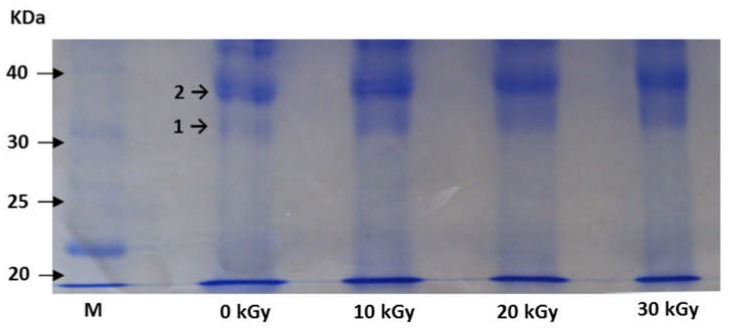
SDS-PAGE gel of samples irradiated at 0, 10, 20 and 30 kGy. M = marker. The bands of interest were identified with numbers 1 and 2. They were removed from the gel and identified by MALDI-TOF/MS..

Samples irradiated at 0, 10, 20 and 30 kGy were submitted to SDS-PAGE in reducing conditions. This technique allowed separation of the analytes of interest from other proteins and impurities. Figure 2 shows the picture of the polyacrylamide gel from the SDS-PAGE obtained after revelation with Coomassie blue. The hydrogenation promoted by the reducing agent DTT caused the breaking of the disulfide bond between RTA and RTB. The main bands of interest were identified with numbers 1 and 2 in Figure 2 according to the crescent order of its respective molecular masses. Band 2, of higher molecular weight, presented approximately double of the length of band 1 in the vertical direction. This observation suggests the possibility of a superposition of the signals of two polypeptide chains, with similar molecular weights, at band 2.

One single band from SDS-PAGE may contain more than one isoform which are differentiated from each other by the degree of glycosylation because differences in the contents of sugar generate small differences in mass [34]. Fultonet et al. [35] and Kim et al. [33], observed that RTA isolated and purified exhibit two bands in the gel revealed with Coomassie blue, while RTB does not present any heterogeneity. Therefore, the whole molecule of ricin splits into two protein bands. The upper band is a mixture of RTB and the first isoform of RTA, while the lower band corresponds exclusively to the second isoform of RTA [33]. All samples presented two bands in the SDS-PAGE gel. The sample irradiated at 30 kGy presented less intense color compared to the others. Considering that the intensity is related to the amount of protein bound to the colorant used, we can deduce that this sample contains the lower concentration of ricin.

In order to make a clear identification, the two bands were collected and analyzed by MALDI-TOF MS. Results (shown in the next topic) allowed the identification of band 1 as one of the isoforms of RTA and band 2 as a mixture between RTB and the second isoform of RTA.

#### 2.2.2. Ricin Identification through MALDI-TOF MS

**Figure 3:**
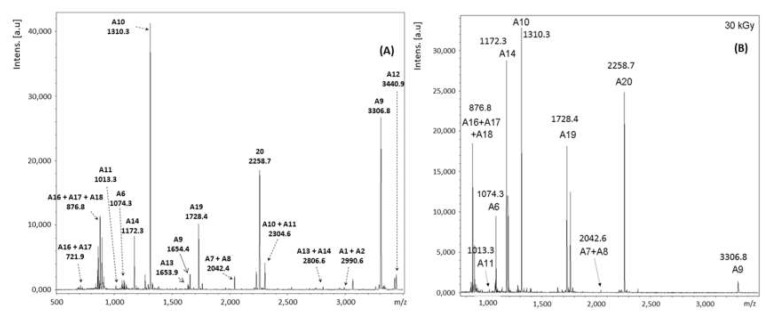
MALDI-TOF MS mass spectra of band 1 (RTA) of the ricin samples. (**A**) Non-irradiated and (**B**) irradiated with 30 kGy.

The two bands in the region of interest in the SDS-PAGE gel were cut, discolored, and digested with trypsin. The peptides obtained this way were extracted from the gel and analyzed through MALDI-TOF MS. Figure 3 shows the result obtained for band 1 of the non-irradiated and the sample irradiated at 30 kGy for comparison purposes.

The mass standard spectrum, also known as the fingerprint of peptides, was used for the identification of the protein. At first, the list of the peak masses was exported to the program Biotools (Bruker^®^). After, through the search mechanism MASCOT PMF, the experimental results were compared to the information available in the data banks: SwissProtb [36] and National Center of Biotechnology Information (NCBI) [37]. The mass standard obtained from the analysis of band 1 showed to be similar to the chain RTA, with the probability of being a random event <0.05.

The radiolysis process may occur directly over the target molecule as a primary effect, or indirectly, through the formation and reaction of free radicals with other molecules present [38]. The gamma irradiation may denature proteins and reduce the amino acids content. Some residues, like the ones containing sulfur, can be more susceptible to radiolysis [39,40]. These phenomena may alter the intensity of peptides peaks as shown in Figure 3.

In Figure 3 the peaks were labeled in order of maintaining the correspondence with the peptides nomenclature shown in Table 2, which presents the expected masses for the complete proteolysis of ricin by trypsin. As not all theoretical scissions occurred, some peaks in Figure 3 were labeled as a sum of peptides, indicating that they remained connected. Table 2 lists the *m*/*z* values measured with the theoretical values of the corresponding peptides, and the respective amino acid sequences and the positions occupied in RTA.

Most of the peptides were identified without modifications, by the mass of their quasi-molecular ion [M + H]^+^, like, for example, A6, A9, A10, A11, A12, A14, A19 and A20. 

The presence of some peaks suggests that the proteolysis was incomplete, producing one single peptide while two or three were expected. This happened for signals at *m*/*z* A1 + A2, A7 + A8, A10 + A11, A13 + A14, A16 + A17 and A16 + A17 + A18.

As the peptide A7 + A8 has an estimated mass of 4083.214 Da, its quasi-molecular ion [A7 + A8 + H]^+^ present *m*/*z* out of the range analyzed (700–3500) and, therefore, cannot be detected. However, it is expected that its doubled protonated ion at *m*/*z* = 2042.615 [A7 + A8 + 2H]^2+^ be detected. This is compatible with the signal observed at *m*/*z* = 2042.4 (see Table 2).

The identification of peptide A13 was not trivial due to the presence of cysteine and methionine residues at positions 171 and 174, respectively. Cysteine residues can react with the acrylamide of the gel, adding 71.04 Da to the mass of the peptide. The signal observed at *m*/*z* = 1653.9 is compatible with this modification. The methionine residue may oxidize to methionine sulfoxide, originating one single peak related to A13. It was possible to identify the presence of this peak together with A14, as a low-intensity peak at 2806.6, corresponding to A13 + A14.

Peptide A14 was also associated with two other peaks at *m*/*z* 1172.2 and 1188.2 (less intense). The difference of 16 units between them can be explained due to the oxidation of the methionine residue at position 188. The same phenomenon explains the peak of peptide A23 at *m*/*z* 2228.5. 

Peptides A3, A4, A15, A16, A17, A18, A21 and A22 were not detected because their masses are <700 Da, and, therefore, out of the range analyzed (700–3500).

The most intense signals of the spectra (Figure 3) were identified as A10 and A9, in descending order. This result is important because these two peptides allow differentiating ricin from the lectin RCA120 also present in castor bean samples. The similarity between the amino acids of RTA and RCA120 is superior to 93%. By comparing the RTBs of both proteins, this value drops to the still high value of 84% [41]. So, in order to eliminate doubts in the identification, it is of fundamental importance to find intense signals of some peptides that allow differentiating ricin from RCA120, like A5, A7, A9, A10, A11, A13, A22, B14, B15, B18, B19 and B20 [42].

Table 3 shows a comparison of the peptide sequences of RTA and RCA120 between positions 86 and 124. The difference between them is at amino acids 114 and 115, underlined in Table 3. While ricin holds an arginine (R) and a tyrosine (Y) at these positions, RCA120 holds a serine (S) and a phenylalanine (F). As trypsin works on the R, the cleavage happens only between amino acids 114 and 115 of ricin. Therefore, only ricin has the peptides A9 and A10, with masses 3307 and 1310 Da. The equivalent sequence of RCA120 has one single peptide with a mass of 4513 Da.

**Figure 4:**
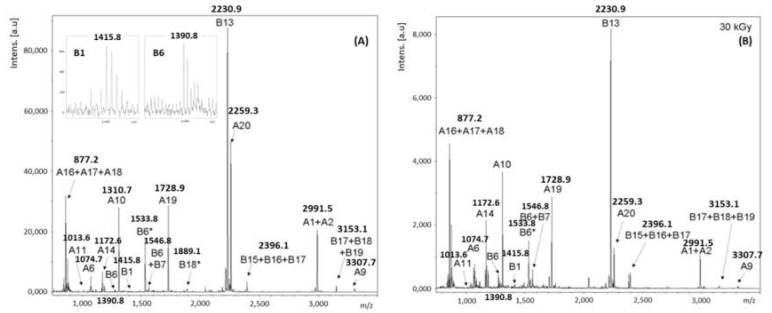
MALDI-TOF MS mass spectra obtained for band 2 of the SDS-PAGE gel of the ricin samples. (**A**) Non-irradiated and (**B**) irradiated with 30 kGy. Magnification of the mass spectra in the region of peptides B1 and B6 is shown in the spectra of the non-irradiated sample.

Due to the relevance of peptides A9 and A10, and the intensity of its peaks in the mass spectra of Figure 4, the ions at *m*/*z* 3307 and 1310, were chosen for confirmation of the sequence of amino acids by MALDI-TOF MS/MS as discussed in the next topic.

MALDI-TOF MS analysis of band 1 of the SDS-PAGE gel identified the presence of RTA in the non-irradiated sample. As band 1 also shows up visible in the SDS-PAGE gels of the irradiated samples, it is reasonable to suppose that RTA is also present in these samples. In fact, this was confirmed by the mass spectra shown in Figure 3 where it is possible to identify the peaks of the selected ions A9 and A10 corresponding to RTA, contrasting with a single peptide for RCA120 (see Table 3).

The main peaks found in band 1 and identified as peptides of RTA, were also observed in the spectra obtained for band 2 of the SDS-PAGE gel for the non-irradiated sample, shown in Figure 4. The main difference was the presence of peptides that compose RTB that were not observed before. Figure 4 and Table 4 present the results of the MALDI-TOF MS analysis of band 2 for the non-irradiated sample. The base ion, located at *m*/*z* 2230.9, corresponds to the peptide B13 (AEQQWALYADGSIRPQQNR).

Besides B13, we also identified B1 and B6 among the peptides expected for RTB. These two, however, presented signals with low intensity when compared to the base ion and, therefore, are magnified in Figure 4. Other three peaks correspond to the clusters B6 + B7, B15 + B16 + B17 and B17 + B18 + B19. Peaks at *m*/*z* 1533.8 and 1889.1 do not belong to ricin but correspond to the peptides B6 * and B18 * of RCA120, being indicatives of a third component in the upper band of the SDS-PAGE gel (Figure 4 and Table 4).

The identification of the peptides present in band 2 was more difficult than for band 1. First due to the presence of different chains interfering in the spectra from each other, and increasing the complexity of the matrix. Besides, several peptides from RTB possess mass values below (B2, B4, B7, B8, B9, B15 e B17) or over (B12) the range of calibration for the method used, and, therefore, could not be identified separately. Finally, even after the addition of a reducing agent (DTT) during sample preparation, some S−S bonds do not break and others may rebind naturally. Therefore, instead of producing one single peptide, many different combinations of fragments with different masses could have happened, making it difficult the identification. A usual alternative to inhibiting the formation of new disulfide bonds after sample reduction is the addition of an alkylating agent, like iodoacetamide or iodoacetic acid, which covalently binds to the thiol group of cysteine. In this case, one should consider the increase in mass due to the addition of this new group.

Despite its major complexity related to band 1, results make it clear that band 2 is composed by the superposition of signals from RTB and one of the isoforms of RTA, corroborating with the literature [33]. Additionally, we also found evidence of the presence of peptides from RCA120. The presence of this contaminant is justified because this is a natural protein of castor bean plants with chains similar to ricin.

**Figure 5:**
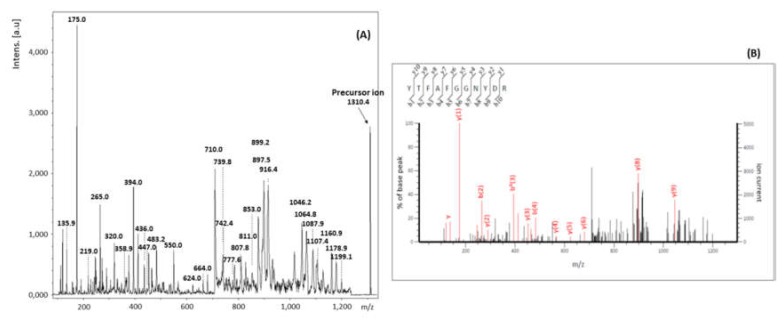
MALDI-TOF MS/MS spectra corresponding to the fragments of precursor ion *m*/*z* 1310 (**A**). Analysis of the MALDI-TOF MS/MS spectra of the precursor ion *m*/*z* 1310 (**B**).

Results of the MALDI-TOF technique analysis of the irradiated samples of band 2 (Figure 5), were very similar to the non-irradiated sample discussed before. The same peptides were identified, and the main difference observed was the intensity reduction of the signals in the spectra compared to the non-irradiated sample. These results show that the use of the technique of MALDI-TOF after separation through SDS-PAGE allowed identification of the presence of ricin in all samples studied, including the ones irradiated at 30 kGy.

#### 2.2.3. Analysis by MALDI-TOF MS/MS

In order to confirm the identification of ricin by a second spectrometric technique, two peptides from RTA and one from RTB were verified by MALDI-TOF MS/MS. The first and second precursor ions selected were the ones with *m*/*z* 1310 Da and 3.307 Da, due to the high intensity of its peaks in the mass spectra, and the relevance of peptides A10 and A9 for the differentiation between ricin and RCA120. The third ion was the one corresponding to B13, with *m*/*z* 2231, for being the most intense related to RTB.

The MALDI-TOF MS/MS spectra corresponding to ion at *m*/*z* 1310 is shown in Figure 5. The data obtained from this spectrum were analyzed through the software Bruker Biotools^®^ (Version 2.2, Bruker Daltonik GmbH, Bremen, Germany), together with the search mechanism MASCOT, and compared to the data banks SwissProt [36] and NCBI [37]. Results were compatible with the amino acids sequence YTFAFGGNYDR, confirming the identification of peptide A10 from ricin.

Table 5 lists the fragments of peptide A10 identified by MALDI-TOF MS/MS. The following ions of series –y and –b of peptide A10, presented correspondence with the products generated from precursor *m*/*z* 1310: y1, y2, y3, y4, y5, y6, y7, y8 and y9; b2, b3 and b4. We also found some ions of the series a (a1, a2 and a7) and immonium, which contributed to reinforcing the interpretation of the results.

**Figure 6:**
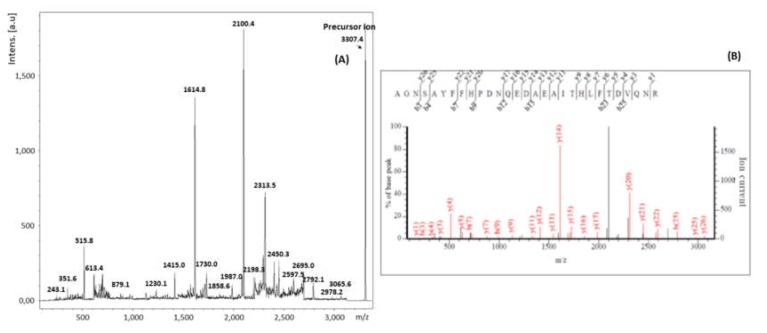
MALDI-TOF MS/MS spectra corresponding to the precursor ion *m*/*z* 3307 (**A**). Analysis of the MALDI-TOF MS/MS spectrum of the precursor ion *m*/*z* 3307 (**B**).

Figure 6 presents the MALDI-TOF MS/MS spectra corresponding to the fragments of ion *m*/*z* 3307, and the corresponding analysis through the software Bruker Biotools^®^ together with the search mechanism MASCOT, and compared to the data banks SwissProt [36] and NCBI [37]. Like before, it is possible to verify that the results are compatible with the amino acid sequence AGNSAYFFHPDNQEDAEAITHLFTDVQNR, confirming the identification of the peptide A9 of ricin. As shown in Table 6, the following series y and b were found: y1, y3, y4, y5, y6, y7, y8, y9, y11, y12, y13, y14, y15, y16, y17, y18, y20, y21, y22, y25 and y26, together with b3, b4, b7, b9, b12, b15, b23 and b25.

**Figure 7:**
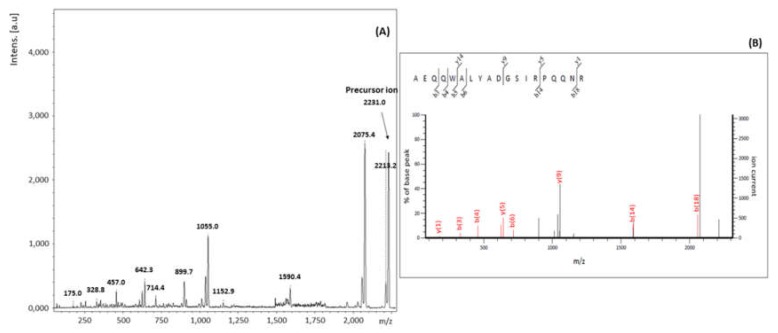
MALDI-TOF MS/MS spectrum corresponding to the fragments of the precursor ion *m*/*z* 2231. (**A**) Analysis of the MALDI-TOF MS/MS spectrum of the precursor ion *m*/*z* 2231 (**B**).

Finally, in order to definitely identify ricin in the samples, the last ion selected for the analysis by MALDI-TOF MS/MS was the *m*/*z* 2231. We tried to verify if the products formed would be compatible with the fragments of peptide B13. Results are shown in Figure 7 and Table 7.

The presence of ions y1, y5, y9 and y14, together with B3, B4, B6, B14 and B18, allowed confirming the similarity between the peak observed in the spectra of *m*/*z* 2331 and the sequence of amino acids of peptide B13 (AEQQWALYADGSIRPQQNR).

#### 2.2.4. Determination of the Toxic Activity by MALDI-TOF MS

The active site responsible for the toxicity of ricin is located in RTA between residues Tyr80 and Trp211. The residues playing the most important role in the mechanism of adenine removal from rRNA 28S are Tyr80, Tyr123, Glu177 and Arg180 [20,43]. This information together with the PMF spectrum obtained by MALDI-TOF MS (Figure 3) allows correlating the ricin activity to peptides A8 to A13. Among them A8, A10 and A13 are the ones containing the most relevant residues [20,43].

All peptides in the region of the active site of ricin were identified by MALDI-TOF MS for both the non-irradiated and the irradiated samples (Figure 3). The presence of these peptides suggests the possibility of toxic activity even in the samples irradiated at 30 kGy.

**Figure 8:**
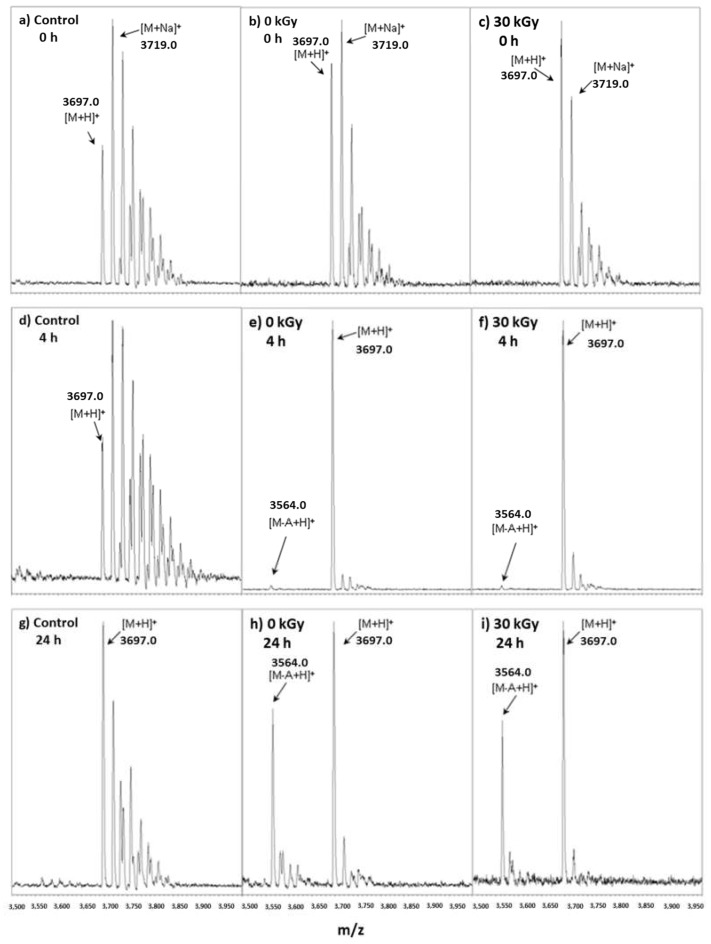
Verification of the toxic activity of ricin by MALDI-TOF MS. (**a**) Control at 0 h; (**b**) Sample 0 kGy at 0 h (**c**) Sample 30 kGy at 0 h; (**d**) Control after 4 h; (**e**) Sample 0 kGy after 4 h (**f**) Sample 30 kGy after 4 h; (**g**) Control after 24 h; (**h**) Sample 0 kGy after 24 h (**i**) Sample 30 kGy after 24 h.

In order to confirm whether the samples presented toxic activity, a non-irradiated sample and another irradiated at 30 kGy, were incubated with a buffer solution containing DNA substrate with a nucleotide sequence similar to rRNA 28S. A buffer solution containing only the DNA substrate was used as a control. Aliquots were collected at three different times of incubation (0, 4 and 24 h) and analyzed by MALDI-TOF MS. Results are shown in Figure 8. At the beginning of the reaction (letters “a”, “b” and “c” in Figure 8), all samples presented a unique set of intense peaks with *m*/*z* values starting at 3697, followed by 3719. These spectra are compatible with the mass of the intact oligonucleotide (GCGCGAGAGCGC) (Figure 8). The first signal corresponds to the quasi-molecular ion [M + H]^+^ and the others to adducts of salts usually present, like sodium salts [M + Na]^+^.

After 4 h of reaction, no alteration was observed in the control sample. However, in the samples incubated with ricin (0 and 30 kGy), it was observed a peak at *m*/*z* 3564 with very low intensity compared to the base peak [M + H]^+^ (Figure 8d,e,f). This same peak became much more intense in the aliquots collected after 24 h of incubation with ricin, reaching around 70% of the intensity of the base peak [M + H]^+^ (Figure 8h,i). The control sample presented only the set [M + H]^+^ and its adducts (Figure 8g). The difference of *m*/*z* between the quasi-molecular ion [M + H]^+^ and the peak at 3564 is of 133 units. This is compatible with the replacement of one adenine base of the nucleotide sequence by a hydrogen atom. The label [M − A + H]^+^ was used to identify this peak in Figure 8.

It was possible to see by MALDI-TOF spectrometry that both samples, the non-irradiated and irradiated at 30 kGy, attacked the DNA substrate, provoking the removal of the adenine nucleotide from the sequence GCGCGAGAGCGC. This result is compatible with the MALDI-TOF MS spectra of the samples where we had already identified the presence of peptides related to the active site of ricin (Figure 3 and Figure 4) and shows that irradiation at 30 kGy is not enough to eliminate totally the toxic activity of ricin.

## 3. Conclusions

Our results showed that the ASE method was efficient and rapid for the extraction of ricin samples from castor bean seeds. For the best of our knowledge, it is the first time that this method is employed to ricin extraction. This method can be improved for future works, including subsequent steps of protein purification, and comparison with other forms of sample preparation reported in the literature [4,14,32,35]. In addition, the use of ASE combined with SDS-PAGE, MALDI-TOF MS and MALDI-TOF MS/MS, has provided a fast and unambiguous identification method for ricin that can be used in real cases of forensic investigation of suspected samples.

The irradiation of samples provoked a strong and gradual reduction in the intensity of the molecular mass signal of ricin measured by MALDI-TOF MS. The signal related to the molecules that remained intact after irradiation at 30 kGy, was so small that it was not possible to distinguish it from the noise. The loss of molecular mass, however, did not imply in the complete destruction of the protein or elimination of the toxicity. Despite the initial results, ricin showed quite resistant to gamma-ray irradiation. This is illustrated by the fact that even after exposure to a dosage of 30 kGy the sample still presented toxic activity, being able to remove the adenine residue from the nucleotide sequence of the DNA substrate. These results can be attributed to two main factors: The first is related to the very low toxicity of ricin already reported by Olsnes [15] who relates that a single unit of RTA is capable of inactivating thousands of ribosomes per minute. This makes any residual remnants of ricin potentially active. The second reason is that probably the mass loss provoked by the irradiation did not alter considerably the active site of the toxin, located in a specific region of RTA. In fact, the principal trypsin peptides of the ricin chains, including the ones related to the toxic activity, were identified in all samples, including the sample irradiated at 30 kGy. 

The SDS-PAGE separation technique used followed by trypsin digestion and analyzed by MALDI-TOF MS, showed an important tool of identification, in this case, making it possible to differentiate ricin from other proteins with similar structures, like the RCA120, for example. Besides, the confirmation by a second method, where the amino acids sequence of the peptides was verified by MALDI-TOF MS/MS, provided higher credibility to the identification.

## 4. Materials and Methods

The materials and methods used for the development of this work are described below. It is important to mention that the manipulation of ricin, even in small amounts, means a huge risk and can cause death by accidental ingestion or inhalation. Besides, the production, storage and using of this toxin are severely restricted by the CWC [9]. 

### 4.1. Protecting Equipment

The samples were produced in a glove box safety cabin equipped with a negative pressure system with HEPA and activated carbon filters, from the chemical biological radiological and nuclear (CBRN) defense Institute of the Brazilian army. Some procedures were also performed in the biology Institute of the Brazilian army in a safety cabin class II. Protective clothes, masks and gloves were needed for most of the experiments.

### 4.2. Sample Preparation

**Figure 9:**
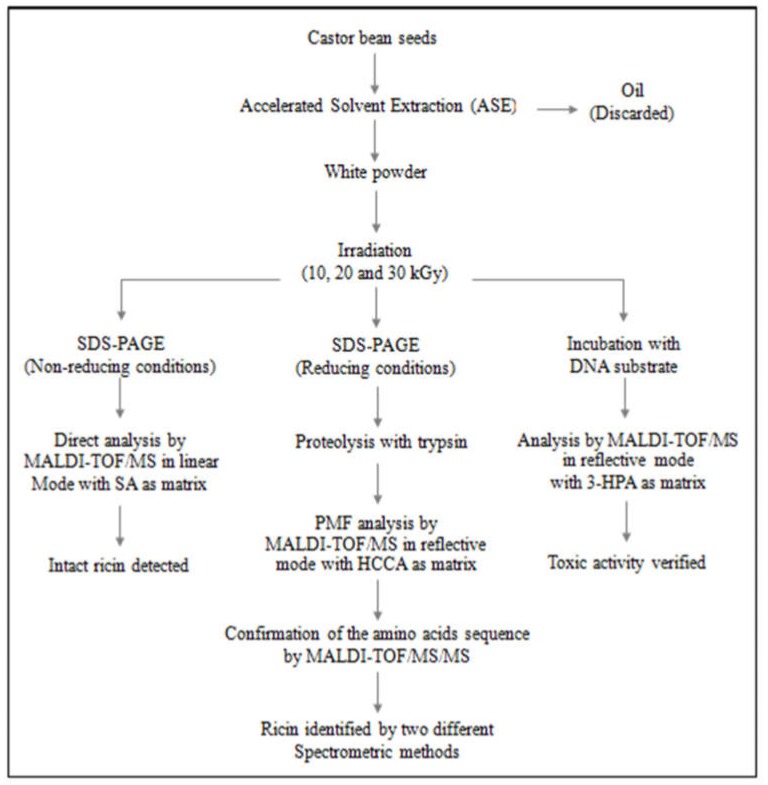
Scheme of preparation and analysis of the ricin samples.

The scheme shown in Figure 9 summarizes all the steps used for the preparation and analysis of the ricin samples used in this work.

### 4.3. Castor Bean Seeds

The castor bean seed used belonged to the species *R. communis* L., cultivar IAC Guarani, harvest 2012/2012, category S2, lot 05/2012, with 96.80% of purity. They were received from the Company “BR Seeds Production and Commerce of Seeds Ltda” based in the city of Araçatuba in the São Paulo State, Brazil.

### 4.4. Production of the Ricin Samples

Samples containing ricin were produced from the seeds of castor bean (*R. communis* L.) adapting the extraction method with acetone described in the literature [34,35,44], for ASE. This was performed in a Dionex extractor (Thermo Fischer Scientific, Waltham, MA, USA), model ASE100, with a 100 mL cylindrical extraction cell made of stainless steel. Firstly, the seeds were peeled with the help of tweezers and a spatula, until exposing their whitish inner part. After, they were milled in an 80 mL Ika^®^ stainless steel knives mill, model A11 from Ika manufacturer. The oiled mass (4 g) was transferred to the extraction cell that was inserted in the oven of the extractor ASE100. The temperature was programmed to stay constant at 40 °C. Extraction was performed with a mixture 8:2 of the solvents n-hexane 99% UV/HPLC-Spectroscopic from Vetec (Rio de Janeiro, Brazil), and acetone 99.8% HPLC from J. T. Baker. This mixture was pumped into the cell, filling the whole volume, and raising the pressure to 1650 psi. After 5 min under this pressure, the extract obtained was filtered and collected into a flask. After removal of the extract, the cell was purged with nitrogen for 1 min and the extraction procedure repeated. After four rounds of extraction and evaporation of the solvent, around 1.2 g of a white powder containing ricin was separated from the extract for each extraction step.

All white powder produced was homogenized and separated in fractions of 1 g. Each fraction was packed into a 15 mL conic tube for centrifugation. After centrifugation, each tube was placed in a transparent plastic bag with a double closing system, identified externally and sent for irradiation. The samples were prepared in triplicate and named according to the irradiation dosages to be received (0 kGy, 10 kGy, 20 kGy and 30 kGy).

### 4.5. Samples Irradiation

The samples were irradiated with gamma rays in the installations of the CBRN Defense Institute of the Brazilian army in a research irradiator of the armored cavity type with a source of Cs^137^, projected and constructed in 1969 in the Brookhaven National Laboratories, in the USA. The source of gamma rays consisted of 28 cylinders of CsCl, with approximately 2.5 cm of length, disposed linearly along a metallic guiding structure. The plastic bags containing the ricin samples were placed over a tray and introduced in one of the two irradiation chambers of the equipment. Samples were irradiated at dosages of 10 kGy, 20 kGy and 30 kGy.

The time of exposure to the irradiation source needed to achieve the desired dosage was calculated through software developed based on the dosimetric mapping of the irradiator. The calculations considered the current activity of the source and the density and geometry of the sample, among other factors. Equation (1) calculates the activity (A) of the source, in kCi·h^−1^, related to the year of the irradiation (t) [45]. 

A = 108·e^−0.23(t − 1969)^ (1)

Based on Equation (1) the value of A for the irradiator in the year of the experiment was of 2.75 Ci·h^−1^. The value found for the dosage absorbed by the samples was of 1.2 kGy·h^−1^. Table 8 lists the time of irradiation necessary for achieving the dosage desired for each sample.

### 4.6. MALDI-TOF MS Analysis

The samples were analyzed in a MALDI-TOF mass spectrometer, Bruker^®^, model microflex LRF. This equipment has a laser of N_2_, with a maximum frequency of 60 Hz, and minimal focus of 50 µm. Each sample was analyzed in triplicate. Firstly, 0.5 µL of solution of sinapinic acid (99% from Sigma-Aldrich (São Paulo, Brazil), saturated in ethanol (95% PA from ACS, Isofar (Duque de Caxias, Rio de Janeiro, Brazil), were applied in one of the spots of a stainless steel target plate. This solution was left to dry and a thin layer of matrix was formed. After, a second solution was prepared, this time containing sinapinic acid saturated in TA30 [30% acetonitrile with 70% water/trifluoroacetic acid (99.9:0.1)]. This solution was mixed in equal parts with a third solution, containing 2 mg/mL of the original sample diluted in 0.1% TFA/water. One aliquot of 0.5 µL of this mixture was collected and applied over the first matrix layer described above, left to dry and analyzed.

The analyses were performed in linear mode by monitoring the presence of peaks in the spectral region corresponding to masses between 50 and 70 kDa. All spectra were acquired by addition after 3000 laser shots, randomly distributed over the whole surface of the sample. The laser energy was kept constant in all shots. 

### 4.7. Inequivocal Identification of Ricin in the Samples

The identification of ricin in the samples was done through MALDI-TOF MS, using the PMF technique, after digestion of RTA and RTB.

#### 4.7.1. SDS-PAGE Under Reducing Conditions

SDS-PAGE of gels were made in an equipment Loccus, model LP3000. The racing and stacking gels were with 12% and 5% (*w*/*v*) of polyacrylamide, respectively. For each sample a 20 mg/mL PBS10 buffer solution (pH 7.4) was prepared. This solution (4 µL) was added to 8 µL of a charging buffer containing the colorant bromophenol blue and other reactants (the commercial product “Blue Loading Buffer Pack”, from BioLabs was used, following instructions of the manufacturer with the addition of the reducing agent dithiothreitol). Finally, 10 µL of the charging solution was applied in one of the spots of a polyacrylamide gel. The bands were removed from the SDS-PAGE gel and identified through MALDI-TOF/MS.

The electrophoresis experiments were performed with a constant tension of 200 V until the migration line achieve 1 cm from the bottom. The gel was immersed in a solution containing ethanol/glacial acetic acid/water (45:10:45) and 1 g of the colorant Coomassie blue for 30 min under gentle stirring. The revelation was done overnight with a solution of methanol/glacial acetic acid/water (3:1:6) at room temperature. Solvents and reagents used were: bright Coomassie blue R250, 98.5%, from Vetec (Rio de Janeiro, Brazil); ethanol 95%, PA, from ACS, Isofar (Duque de Caxias, Rio de Janeiro, Brazil); glacial acetic acid 99.8%, PA, from ACS, Proquímios; methanol 100%, from ACS, J.T. Baker; and distilled and deionized water produced in the lab.

#### 4.7.2. Proteolytic Digestion

After revelation of the SDS-PAGE gel, each band related to the ricin chains was cut out with a stiletto and transferred to a 1 mL microcentrifuge tube, pre-washed twice with TA50 [50% acetonitrile with 50% water/trifluoroacetic acid (99.9:0.1)]. After, the samples were uncolored through two successive washes with 0.2 mL of a solution 100 mM of NH_4_HCO_3_/50% ACN, for 45 min at 37 °C. Then the samples were dehydrated by adding 100 µL of acetonitrile [99.9%, UV/HPLC spectroscopic from Vetec (Rio de Janeiro, Brazil)] for 10 min at room temperature, and dried under N_2_ flow at room temperature.

In parallel, aliquots were collected from the stock solution of trypsin (trypsin gold, mass spectrometry grade from Promega (Madison, Wisconsin, USA) 1 µg/µL in 50 mM of acetic acid, and diluted to 20 µg/mL with 40 mM NH_4_HCO_3_/10% ACN. The dried gel pieces were then incubated and rehydrated in 30 µL of this trypsin solution at room temperature for 1 h. After, the digestion buffer (40 mM NH_4_HCO_3_/10% ACN) was added until covering completely the gel pieces. The tubes were well closed to avoid evaporation and incubated overnight at 37 °C. The day after the solution was transferred to a clean tube and 30 µL of TA50 added to the gel, which was submitted to ultrasound for 20 minutes. The resulting solution was transferred to a clean tube and totally dried under N_2_ (AP, 99.997% from Linde) flow, being re-suspended again with 20 µL of the solution of TFA 0.1% in water. This sample, containing the peptides from the trypsin digestion was sent for analysis by MALDI-TOF MS.

Table 9 shows the peptides expected for the complete trypsinization of ricin, named according to its positions in the sequences of RTA and RTB.

#### 4.7.3. MALDI-TOF MS Analysis for the Identification of Ricin

The mixture of trypsin peptides extracted from each band of the SDS-PAGE gel was analyzed through MALDI-TOF MS using a saturated solution of 4-hydroxy-α-cyanocinnamic acid (HCCA) saturated in TA30 as a matrix. The sample preparation consisted of mixing equal volumes of the peptides solution in 0.1% TFA/water with a saturated solution of HCCA in TA30. After, 0.5 µL of this new mixture was applied over the target plate and left to dry. The analyses were performed in reflector mode, by monitoring the presence of peaks in the spectral region corresponding to the weight between 700 and 4000 Da. All spectra were obtained by addition of 2000 laser shots randomly distributed over the whole surface of the sample. The laser energy was kept constant during the shots.

The mass spectra of the mixture of peptides were used for the identification of ricin. Firstly, the *m*/*z* list of the peaks obtained was exported for the software Biotools, from Bruker. After, through the MASCOT PMF search mechanism, the experimental results were compared with the information available in the data banks of proteins SwissProt [36] and NCBI [37].

#### 4.7.4. Analyses by MALDI-TOF MS/MS

The results obtained by MALDI-TOF MS were confirmed by a second analytic spectrometric technique. For this, three peptides were chosen to have their amino acid sequences verified through MALDI-TOF MS/MS. The criteria established for the selection of the precursor ions were the intensity and the relevance of the peptide for the differentiation between ricin and other proteins, the absence of cysteine and methionine residues, and the possibility of the same peptide representing RTA and RTB.

The analyses were performed in the same target plate with the samples former prepared for the MALDI-TOF MS experiment. The equipment used also was the same used before. The spectra were obtained by the method known as fragmentation analysis and structural time of flight (FAST), which only works in the reflective mode. For each analysis the range of the ions selector and the number of segments were adjusted according to the mass of the precursor ion selected, avoiding interference of fragments from possible adjacent ions. The spectra were exported to the software Bruker Biotools and, with the help of the MASCOT searching mechanism, compared to the data existing in the data banks SwissProt [36] and NCBI [37].

### 4.8. Verification of the Toxic Activity of the Ricin Samples by MALDI-TOF MS

The toxic activity of ricin present in the samples was verified by MALDI-TOF MS, following a method adapted from Schieltz et al. [46]. For this, a DNA substrate chemically synthesized with the nucleotide sequence GCGCGAGAGCGC, similar to rRNA 28S where the ricin attack occurs, was acquired from the Company Genone Biotechnologies (Rio de Janeiro, Brazil).

A solution containing 0.1 µmol/mL of nucleotides was prepared and mixed with a PBS10 buffer (pH 7.4). After, the sample solution was prepared with 20 mg/mL of the white powder extracted from castor bean seeds mixed with the PBS10 buffer solution. The reaction mixture was produced by mixing equal volumes of the two solutions. Then it was incubated at 37 °C, without stirring, for 24 h. Aliquots were collected and analyzed in times 0, 4 and 24 h. The matrix solution consisted of 3-hydroxypicolinic acid (3-HPA) saturated in TA50. This solution (0.5 µL) was applied over the target plate and left to dry at room temperature. At the time intervals mentioned above, 2.0 µL were collected from the supernatant of the reaction and mixed with more 18 µL of the matrix solution. From this mixture, one aliquot of 0.5 µL was deposited over the first layer, left for drying, and introduced in the target plate of the equipment.

The analysis method was in the reflective mode, with a range of *m*/*z* from 3000 to 4000, with the addition of spectra obtained after 2000 laser shots randomly distributed over the whole sample surface in the target plate.

We monitored the intensities of the signals of peaks at *m*/*z* 3,697, referring to the mass of the quasi-molecular ion of the oligonucleotide protonated [M + H]^+^ and in *m*/*z* 3564, related to the loss of adenine [M+H-A]^+^.

Solvents and reagents used for these experiments were: 3-hydroxypicolinic acid 99%, from Sigma Aldrich, TA50 (produced with acetonitrile 99,9%, UV/HPLC spectroscopic, from Vetec (Rio de Janeiro, Brazil); trifluoroacetic acid 99% from Sigma Aldrich and distilled and deionized water); PBS10 (prepared with Na_2_HPO_4_ 99% from Sigma Aldrich; NaH_2_PO_4_·H_2_O 98% from Sigma Aldrich and NaCl, ACS reagent, from Vetec (Rio de Janeiro, Brazil).

## Figures and Tables

**Table 1:** Ratio signal to noise (S/N) of ricin in the samples for each irradiation dosage (average values, three repetitions).

Irradiation Dosage (kGy)	Intensity Signal/Noise (S/N)	(S/N of Sample)/ (S/N of the Non-Irradiated Sample) (%)
0	18.1	100.00
10	6.2	34.12
20	1.5	8.19
30	0.5	2.53

**Table 2:** Ricin peptides identified by MALDI-TOF MS in the band 1 of the non-irradiated sample.

Peptides	Positions	Amino Acids Sequence	[M + H]^+^ Theoretical	*m*/*z* Measured
A1 + A2	1–26	IFPKQYPIINFTTAGATVQSYTNFIR	2990.577	2990.6
A6	40–48	HEIPVLPNR	1074.605	1074.3
A7 + A8	49–85	VGLPINQRFILVELSNHAELSVTLALDVTNAYVVGYR	4084.222	2042.4 ^a^
A9	86–114	AGNSAYFFHPDNQEDAEAITHLFTDVQNR	3307.504	3306.8
				1654.4 ^a^
A10	115–125	YTFAFGGNYDR	1310.580	1310.3
A11	126–134	LEQLAGNLR	1013.574	1013.3
A10 + A11	115–134	YTFAFGGNYDRLEQLAGNLR	2305.136	2304.6
A12	135–166	ENIELGNGPLEEAISALYYYSTGGTQLPTLAR	3440.722	3440.9
A13	167–180	SFIICIQMISEAAR	1652.849 ^b^	1653.9
A13 + A14	167–189	SFIICIQMISEAARFQYIEGEMR	2806.370 ^b^	2806.6
A14	181–189	FQYIEGEMR	1172.540	1172.3
A14	181–189	FQYIEGEMR	1188.535 ^c^	1188.2
A16 + A17	192–196	IRYNR	721.410	721.9
A16 + A17 + A18	192–197	IRYNRR	877.512	876.8
A19	198–213	SAPDPSVITLENSWGR	1728.855	1728.4
A20	214–234	LSTAIQESNQGAFASPIQLQR	2259.173	2258.7
A23	240–258	FSVYDVSILIPIIALMVYR	2228.240 ^c^	2228.5

^a^ Value corresponding to the double protonated ion. ^b^ Considering the formation of the polyacrylamide adduct; ^c^ Considering oxidation of methionine to methionine sulfoxide.

**Table 3:** Comparison between the amino acid sequences of RTA and RCA120 between positions 85 and 126 *.

Protein	Amino Acid Sequences between Positions 85 and 126 of Chain A for Ricin and RCA120
Ricin	**...R^85^AGNSAYFFHPDNQEDAEAITHLFTDVQNRYTFAFGGNYDRL^126^...**
RCA120	**...R^85^AGNSAYFFHPDNQEDAEAITHLFTDVQNSFTFAFGGNYDRL^126^...**

* Different amino acids in both sequences are underlined. The digestion with trypsin leads to peptides A9 (in red) and A10 (in blue) in ricin but keeps RCA120 as a single peptide (in green).

**Table 4:** Peptides identified by MALDI-TOF MS in band 2 of the non-irradiated sample.

Peptides	Positions	Amino Acids Sequence	[M + H]^+^ Theoretical	*m*/*z* Measured
A1 + A2	1–26	IFPKQYPIINFTTAGATVQSYTNFIR	2990.577	2991.5
A16 + A17 + A18	192–197	IRYNRR	877.512	877.2
A6	40–48	HEIPVLPNR	1074.605	1074.7
A7 + A8	49–85	VGLPINQRFILVELSNHAELSVTLALDVTNAYVVGYR	4084.222	2043.0
A9	86–114	AGNSAYFFHPDNQEDAEAITHLFTDVQNR	3307.504	3307.7
A10	115–125	YTFAFGGNYDR	1310.580	1310.7
A11	126–134	LEQLAGNLR	1013.574	1013.6
A14	181–189	FQYIEGEMR	1172.540	1172.6
A19	198–213	SAPDPSVITLENSWGR	1728.855	1728.9
A20	214–234	LSTAIQESNQGAFASPIQLQR	2259.173	2259.3
A23	240–258	FSVYDVSILIPIIALMVYR	2228.240 ^a^	2229.1
B1	1–12	ADVCMDPEPIVR	1415.666	1415.8
B6	41–52	SNTDANQLWTLK	1390.696	1390.8
B6 + B7	41–53	SNTDANQLWTLKR	1546.797	1546.8
B13	169–182	AEQQWALYADGSIRPQQNR	2231.095	2230.9
B15 + B16 + B17		ETVVKILSCGPASSGQRWMF K	2395.226	2396.1
B17 + B18 + B19	216–243	WMFKNDGTILNLYSGLVLDVRASDPSLK	3152.645	3153.1
B6 *	RCA120	SNTDWNQLWTLR	1533.744	1533.8
B18 *		NDGTILNLYNGLVLDVR	1889.013	1889.1

^a^ Considering the oxidation of methionine to methionine sulfoxide. * Peptide of RCA120.

**Table 5:** Fragments of peptide A10 identified by MALDI-TOF MS/MS.

Ions	Amino Acid Sequences	*m*/*z* Theoretical	*m*/*z* Measured
precursor	YTFAFGGNYDR	1310.580	1310.4
b2	YT	265.118	265.0
b3	YTF	412.187	412.0
b4	YTFA	483.224	483.2
y1	R	175.119	175.0
y2	DR	290.146	290.0
y3	YDR	453.209	453.0
y4	NYDR	567.252	567.1
y5	GNYDR	624.274	623.9
y6	GGNYDR	681.295	680.8
y7	FGGNYDR	828.363	828.1
y8	AFGGNYDR	899.401	899.2
y9	FAFGGNYDR	1046.469	1046.2

**Table 6:** Fragments of peptide A9 (AGNSAYFFHPDNQEDAEAITHLFTDVQNR) identified by MALDI-TOF MS/MS.

Ions	Corresponding Amino Acids Sequence	*m*/*z* Theoretical	*m*/*z* Measured
precursor	AGNSAYFFHPDNQEDAEAITHLFTDVQNR	3307.504	3307.4
b3	AGN	243.109	243.1
b4	AGNS	330.141	329.7
b7	AGNSAYF	711.310	711.3
b9	AGNSAYFFH	995.437	995.2
b12	AGNSAYFFHPDN	1321.560	1321.7
b15	AGNSAYFFHPDNQED	1693.688	1693.8
b23	AGNSAYFFHPDNQEDAEAITHLF	2576.148	2576.6
b25	AGNSAYFFHPDNQEDAEAITHLFTD	2792.222	2792.1
y1	R	175.119	175.5
y3	QNR	417.220	417.1
y4	VQNR	516.289	515.8
y5	DVQNR	631.316	631.0
y6	TDVQNR	732.363	732.0
y7	FTDVQNR	879.432	879.1
y8	LFTDVQNR	992.516	992.7
y9	HLFTDVQNR	1129.575	1129.4
y11	ITHLFTDVQNR	1343.707	1343.5
y12	AITHLFTDVQNR	1414,744	1415.0
y13	EAITHLFTDVQNR	1543.786	1544.1
y14	AEAITHLFTDVQNR	1614.823	1614.8
y15	DAEAITHLFTDVQNR	1729.850	1730.0
y16	EDAEAITHLFTDVQNR	1858.893	1858.7
y17	QEDAEAITHLFTDVQNR	1986.952	1987.0
y18	NQEDAEAITHLFTDVQNR	2100.994	2100.4
y20	PDNQEDAEAITHLFTDVQNR	2313.074	2313.5
y21	HPDNQEDAEAITHLFTDVQNR	2450.133	2450.3
y22	FHPDNQEDAEAITHLFTDVQNR	2597.202	2597.5
y25	AYFFHPDNQEDAEAITHLFTDVQNR	2978.370	2978.2
y26	SAYFFHPDNQEDAEAITHLFTDVQNR	3065.402	3065.6

**Table 7:** Fragments of peptide B13 (AEQQWALYADGSIRPQQNR) identified by MALDI-TOF MS/MS.

Ions	Corresponding Amino Acids Sequence	*m*/*z* Theoretical	*m*/*z* Measured
precursor	AEQQWALYADGSIRPQQNR	2231.095	2231.0
b3	AEQ	329.146	328.8
b4	AEQQ	457.204	457.0
b6	AEQQWA	714.321	714.4
b14	AEQQWALYADGSIR	1589.771	1589.7
b18	AEQQWALYADGSIRPQQN	2056.984	2057.3
y1	R	175.119	175.0
y5	PQQNR	642.332	642.3
y9	GSIRPQQNR	1055.570	1055.3
y14	ALYADGSIRPQQNR	1588.819	1588.9

**Table 8:** Time needed for each sample.

Irradiation Dosage Absorbed	Exposure Time
10 kGy	8 h 20 min
20 kGy	16 h 40 min
30 kGy	25 h 00 min

**Table 9:** Expected peptides from the total proteolysis of ricin with trypsin.

Abbreviation	Position	Sequence of Amino Acids	Molecular Mass (M)
A1	36–39	IFPK	504.3
A2	40–61	QYPIINFTTAGATVQSYTNFIR	2504.3
A3	62–64	AVR	344.2
A4	65–66	GR	231.1
A5	67–74	LTTGADVR	831.4
A6	75–83	HEIPVLPNR	1073.6
A7	84–91	VGLPINQR	895.5
A8	92–120	FILVELSNHAELSVTLALDVTNAYVVGYR	3205.7
A9	121–149	AGNSAYFFHPDNQEDAEAITHLFTDVQNR	3306.5
A10	150–160	YTFAFGGNYDR	1309.6
A11	161–169	LEQLAGNLR	1012.6
A12	170–201	ENIELGNGPLEEAISALYYYSTGGTQLPTLAR	3439.7
A13	202–215	SFIICIQMISEAAR	1580.8
A14	216–224	FQYIEGEMR	1171.5
A15	225–226	TR	275.2
A16	227–228	IR	287.2
A17	229–231	YNR	451.2
A18	232–232	R	174.1
A19	233–248	SAPDPSVITLENSWGR	1727.9
A20	249–269	LSTAIQESNQGAFASPIQLQR	2258.2
A21	270–270	R	174.1
A22	271–274	NGSK	404.2
A23	275–293	FSVYDVSILIPIIALMVYR	2211.2
A24	294–302	CAPPPSSQF	932.4
B1	315–326	ADVCMDPEPIVR	1343.6
B2	327–330	IVGR	443.3
B3	331–338	NGLCVDVR	874.4
B4	339–341	DGR	346.2
B5	342–354	FHNGNAIQLWPCK	1526.8
B6	355–366	SNTDANQLWTLK	1389.7
B7	367–367	R	174.1
B8	368–372	DNTIR	617.3
B9	373–376	SNGK	404.2
B10	373–376	CLTTYGYSPGVYVMIYDCNTAATDATR	2948.3
B11	404–416	WQIWDNGTIINPR	1611.8
B12	417–482	SSLVLAATSGNSGTTLTVQTNIYAVSQGWLPTNNTQPFVTTIVGLYGLCLQANSGQVWIEDCSSEK	6932.4
B13	417–482	AEQQWALYADGSIRPQQNR	2230.1
B14	502–512	DNCLTSDSNIR	1236.5
B15	513–517	ETVVK	574.3
B16	518–529	ILSCGPASSGQR	1174.6
B17	530–533	WMFK	610.3
B18	534–550	NDGTILNLYSGLVLDVR	1861.0
B19	551–557	ASDPSLK	716.4
B20	558–576	QIILYPLHGDPNQIWLPLF	2276.2
